# Foreign Bodies in the System

**Published:** 1845-06

**Authors:** 


*Foriegn Bodies in the System.*—Mr. Chance detailed the case of a patient who came under his care some months ago with a wound in the arm, which he stated to have been produced by a liniment, which was ordered for rheumatic pains, under which he was laboring. Upon close investigation, it was discovered that some time previous to his suffering, he had run a piece of glass into his arm. He, Mr. Chance, cut down upon the most tender part, and extracted a piece of glass, one inch in length, and half an inch broad at the base, which lay between the tendons of the flexor sublimis and profundus. About a month afterwards, a blacksmith came to him with a piece of steel about half an inch square, which had been imbedded in his arm for about three years. Upon bending the hand upon the arm, a swelling was perceptible. The case was attended with pains of a rheumatic character. The swelling was cut down upon, and the piece of steel removed. He suffered from no pain afterwards. Mr. Chance enquired of members the reason of abscess following, in some cases, the introduction of foreign bodies into the lining tissues, and not in others, and also how the pains so closely resembling rheumatism could be explained.

Mr. Fisher related a case in which a bullet passed from the maxillary glands to the middle of the neck, and which when removed by himself, after a considerable period, was found to be coated with phosphate of lime. In answer to a question, Mr. Chance observed, that the parts surrounding the foreign bodies in his cases were perfectly healthy; there was no sac and no condensation of cellular tissue.

Mr. Hird remarked that, as a general rule, inflammation took place around the foreign body, and a sac was formed.

Mr. Fisher related the case of a man who fell from a ladder through a skylight. He cut himself much, but very speedily recovered. About eighteen months after the accident, however, he complained of great pain in the palmar fascia. Mr. Fisher made an incision into this part, and extracted three pieces of glass.

Dr. Ried recollected a case which occurred in St. Giles's Infirmary. The patient complained of great pain in the knee, on the side of which there was a moveable swelling, closely resembling a loose cartilage. An incision being made into it, a large darning-needle, much bent, and black, was extracted. The patient then remembered that it had passed in fifteen months previously.

Dr. Sayer related the case of a lady, forty-eight years of age, who accidentally received a pistol-bullet between the fifth and sixth ribs. It passed between these bones without injuring them, or wounding the intercostal artery: it passed into the thoracic cavity, and there remained for several years. Occasionally, and particularly on the least exertion, she was subjected to distressing attacks, bearing every similitude to spasmodic asthma. The immediate effect of the injury had been the production of pneumonia, which, however, gave way under bleeding, and other antiphlogistic remedies. On examination after death, the right lung was found healthy, but the lung corresponding to the wound had become adherent by a pouch to the thoracic parietes. In this pouch was found a bullet. He recollected the case of a young lady in whom severe pain came on suddenly whilst she was riding, in the upper part of the thigh. She was obliged to be carried home. Upon examination of the part nothing could be detected, but she experienced considerable pain on pressure behind the upper part of the trochanter, and on an incision being made into this part, a needle, an inch and a half long, black, and coated here and there with oxide of iron, was extracted. She had no recollection of the time or manner in which the needle had become introduced into the system.—*London Lancet.*

